# Successful diagnosis of asymptomatic choriocarcinoma by ultrasonography during a viable pregnancy: A rare case report

**DOI:** 10.1097/MD.0000000000037334

**Published:** 2024-03-01

**Authors:** Qianqian Gao, Hong Luo

**Affiliations:** a Department of Ultrasound, West China Second University Hospital, Sichuan University, Chengdu, Sichuan, P.R. China; b Key Laboratory of Obstetric & Gynecological and Pediatric Diseases and Birth Defects of Ministry of Education, Sichuan University, Chengdu, Sichuan, P.R. China.

**Keywords:** choriocarcinoma, ultrasound, viable pregnancy

## Abstract

**Background:**

Gestational choriocarcinoma occurs very rare in conjunction with pregnancy and it is camouflage for diagnosis.

**Methods:**

We present a rare case of asymptomatic choriocarcinoma in a viable pregnancy that was successfully diagnosed by ultrasonography and had timely treatment.

**Results:**

According to the ultrasonography, early diagnosis and treatment monitoring of choriocarcinoma during a viable pregnancy was administered and the newborn was discharged.

**Conclusion:**

Choriocarcinoma in pregnancy is camouflaged, and its clinical presentation varies widely. Despite an asymptomatic status, metastasis can occur, and ultrasonography is important for early diagnosis and treatment monitoring.

## 1. Introduction

Gestational trophoblastic neoplasia (GTN) refers to lesions that have the potential for local invasion and metastasis, including the invasive mole, choriocarcinoma, placental site trophoblastic tumor, and epithelioid trophoblastic tumor.^[1]^ Depending on the origination, choriocarcinoma is divided into gestational choriocarcinoma and nongestational choriocarcinoma.^[2]^ Gestational choriocarcinoma occurs in approximately 1 in 20,000 to 40,000 pregnancies^[1]^ and even less frequently in conjunction with pregnancy. Although choriocarcinoma with a concurrent viable pregnancy is very rare, choriocarcinomas, including intraplacental choriocarcinoma (IC) and extragenital choriocarcinoma, can be found in areas other than the uterus.^[3,4]^ When choriocarcinoma occurs during viable pregnancy, there are often no obvious symptoms in the early stage. When it does present with maternal symptoms such as vaginal bleeding or hemoptysis, it has already reached the late stage.^[5]^ Ultrasonography (US) and magnetic resonance imaging (MRI) are often used to diagnose uterine masses. On grayscale US, choriocarcinoma may be hyperechoic or hypoechoic and homogeneous or heterogeneous and may show small anechoic cystic spaces; on color Doppler US, the mass is usually highly vascularized; and on spectral Doppler US, trophoblastic vessels demonstrate a high-velocity low-resistance waveform.^[5]^ However, the appearance of GTN on MRI is nonspecific, and is limited to differentiate between the various types of GTN.^[5,6]^ Here, we present a case of choriocarcinoma with a concurrent viable pregnancy at 18 weeks of gestation that was diagnosed by US. Written informed consent was obtained from the patient for publication of this case report and any accompanying images.

## 2. Case presentation

**Figure 1.**
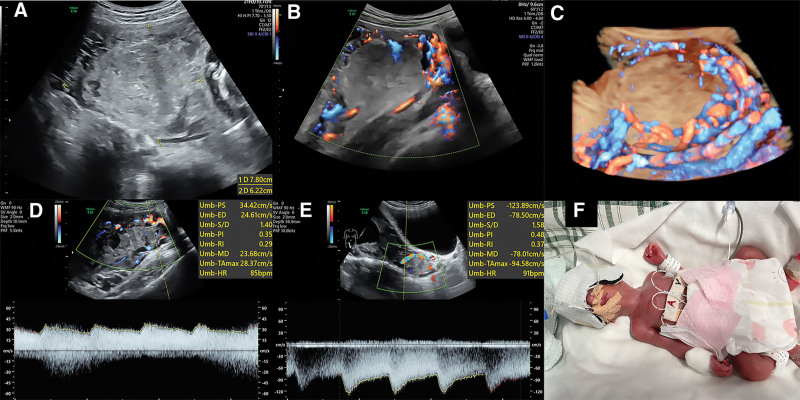
(A) The complex mass with solid and cystic components was on the right posterior uterine wall. (B) Multiple turbulent flows, consisting of a tangle of vessels with multidirectional high-velocity flow, were evident in and around the mass. (C) The three-dimensional color Doppler image of the mass showed hypervascularity in and around the lesion. (D) Spectral Doppler showed the low-resistance blood flow spectrum, the peak systolic velocity (PSV) was 34.42 cm/s, the resistive indexes (RI) was 0.29, and the pulsatility index (PI) was 0.35. (E) The uterine artery pulsatility index (UAPI) was 0.48 and RI was 0.37. (F) The newborn image of 26^+4^ weeks gestational age at birth. PSV = peak systolic velocity, PI = pulsatility index, RI = resistive indexes, UAPI = uterine artery pulsatility index.

A 27-year-old woman, gravida 2, para 0, had a uterine mass found at 18 weeks’ gestation and underwent a mid-trimester ultrasound scan using the General Electric Healthcare Voluson E10 (Zipf, Austria) with C4-8-D transducer. The woman did not have any symptoms. The US examination showed a normal fetus and a 6.5 × 6.7 × 7.6-cm complex mass on the right posterior uterine wall (Fig. 1A). Multiple turbulent flows were found in and around the mass (Fig. 1B). Around the mass, the uterine myometrium wall was very thin and seemingly retained only uterine serosa. In addition, the boundary of the mass was unclear and the shape was irregular. The three-dimensional color Doppler image of the mass showed hypervascularity in and around the lesion (Fig. 1C) and spectral Doppler showed the low-resistance blood flow spectrum (Fig. 1D). Uterine blood vessels were also dilated and the uterine artery pulsatility index (UAPI) was 0.48 (Fig. 1E). One week later, the mass grew to 7.8 × 6.2 × 8.3 cm, but none of the other characteristics changed. Based on the ultrasound image, GTN was highly suspected. The β-human chorionic gonadotropin (β-hCG) level ranged to 156,004 mIU/mL at 18 weeks gestation. Subsequently, a review of the patient’s medical history revealed that she was diagnosed with complete hydatidiform mole and invasive mole 4 years ago, and the lesion was also in the right uterine myometrium. After 6 rounds of chemotherapy, the β-hCG status became negative, and the lesion disappeared. Given the patient’s strong wish to continue her pregnancy, her genital tract was confirmed to be normal by gynecological examination, and the uterine mass, lung, and brain was further evaluated by MRI. Unfortunately, lung metastasis was found and confirmed by a pathologist. Finally, considering the gestational age, the patient’s willingness to continue the pregnancy, and the risk of termination of pregnancy, immediate chemotherapy was recommended. Then, the patient accepted chemotherapy with etoposide, methotrexate, actinomycin D, cyclophosphamide, and vincristine. Twenty days later, the third round of chemotherapy was carried out. However, the fetal growth restriction (FGR) was diagnosed (the estimated fetal weight was <1%). At 26^+4^ weeks of gestation, the patient underwent the fourth round of chemotherapy, but an inevitable abortion was experienced, a male newborn was delivered and the birth weight was 637 g (Fig. 1F). Then the newborn was transferred to the neonatal intensive care unit for treatment and discharged 4 months later with the weight of 2520g. After delivery, the patient continued chemotherapy and had hysterectomy 4 months later.

## 3. Discussion

Gestational choriocarcinoma is extremely rare in a viable pregnancy. It can be detected several months after an identifiable gestational event^[7]^ and has a long incubation period that last up to several decades.^[5]^ In addition, choriocarcinoma easily invades blood vessels and metastasizes at an early stage. Most of the lesions located in the myometrium and may soon invade other organs, such as the parametrium, vagina, other pelvic organs, and lung.^[7]^ In our case, the patient was diagnosed with complete hydatidiform mole and invasive mole 4 years ago and clinically cured. At gestation, although she was asymptomatic, lung metastasis was diagnosed. The primary site of choriocarcinoma with a concurrent viable pregnancy can be in the uterine, intraplacental, or extragenital region due to its microscopic nature.^[2]^ In cases where the choriocarcinoma occurs in a viable pregnancy, one can presume that the choriocarcinoma arose either from the concurrent pregnancy or from the transformation of retained tissue from a previous pregnancy.^[7]^ Because the lesion was in the same uterine myometrial wall as the previous pregnancy, we concluded that the invasive mole lesion was probably the etiology of the choriocarcinoma.

The hCG level is a useful marker for both pregnancy and choriocarcinoma,^[8]^ and in general, levels of hCG rise steadily during the first 30 to 45 days of normal pregnancy but only rarely exceed 10^5^ to 2 × 10^5^.^[1,8,9]^ In contrast, hCG levels observed with choriocarcinoma are directly related to the number of tumor cells.^[1,8,9]^ If β-hCG levels continue to rise or do not decrease to an acceptable level postpartum or postabortion, choriocarcinoma should be considered.^[1]^ Despite these general guidelines, hCG levels should not be used as an independent measure of choriocarcinoma.^[8,9]^ Nevertheless, it does provide an excellent marker for the measurement of chemotherapy response in choriocarcinoma.^[8]^

US is the modality of choice for the initial diagnosis of GTN, and it can provide an invaluable means of local surveillance after treatment. US images lack specificity for the diagnosis of GTN, but it is helpful for detecting a mass.^[5]^ Usually, GTN are seen at grayscale US as focal masses centered within the myometrium with a variable endometrial component.^[5]^ The focal masses of choriocarcinoma may be hyperechoic or hypoechoic and homogeneous or heterogeneous and may show small anechoic cystic spaces on grayscale US.^[5]^ The lesions are “dome-shaped” or “gourd-shaped” as they connect with the uterus, the boundaries of the lesions are unclear, and the mass can reach or even pass through the serosal layer.^[6]^ On color Doppler US, abnormally rich blood flow was found in the lesion and indicated high vascularization in the mass due to intralesional arteriovenous shunts.^[5]^ In addition, on spectral Doppler US, trophoblastic vessels demonstrated a high-velocity low-resistance wave form, and significantly lower resistive indexes (RIs) (0.25) were present for choriocarcinoma.^[5]^ UAPI is inversely proportional to tumor vascularity, and a low UAPI is indicative of increased uterine blood flow and arteriovenous shunting.^[5]^ Due to the nonspecificity of US in the diagnosis of choriocarcinoma, accurate diagnosis depends on correlation with clinical findings and β-hCG levels. US was used to monitor the maximal diameter of the uterine mass, shape, depth of muscle invasion and serosal layer continuity.^[5]^ Instead of US, MRI is often used to evaluate uterine masses. As with US, MRI will not be better for the specific diagnosis of GTN. However, MRI is superior to US in the evaluation of extrauterine pelvic tumor extension and pelvic lymph nodes.^[5,6]^

According to the literatures, choriocarcinoma is sensitive to chemotherapy with etoposide, methotrexate, actinomycin D, cyclophosphamide, and vincristine, but the regimen can increase the likelihood of perinatal complications such as fetal growth restriction.^[7,9,10]^ However, the survival rate depends on the initiation time of therapy. Survival is reported to be 90% with chemotherapy, while untreated patients face a median survival of <4 months.^[9]^ Metastatic spread of choriocarcinoma during a viable pregnancy is common, and often with poor prognosis may be related to late stage at the time of diagnosis.^[7,9]^ In our case, although the patient did not have any symptoms, a uterine mass was found during the routine obstetric examination, and lung metastasis was diagnosed. According to the immediate chemotherapy, the newborn was alive and the patient was cured.

## 4. Conclusion

Choriocarcinoma during a viable pregnancy is extremely rare but is camouflaged. As it is difficult to diagnose, it must be considered when an intermural mass of the uterine muscle is identified in a pregnant woman. US is the modality of choice for the initial diagnosis of pregnancy and GTN, and it can also provide an invaluable means of local surveillance after treatment. Although a patient is asymptomatic, metastasis may also occur, and early diagnosis and treatment are important for survival.

## Author contributions

**Resources:** Hong Luo.

**Validation:** Qianqian Gao.

**Writing – original draft:** Qianqian Gao.

**Writing – review & editing:** Hong Luo.
